# Signal Transduction in Plant–Nematode Interactions

**DOI:** 10.3390/ijms19061648

**Published:** 2018-06-02

**Authors:** Muhammad Amjad Ali, Muhammad Shahzad Anjam, Muhammad Amjad Nawaz, Hon-Ming Lam, Gyuhwa Chung

**Affiliations:** 1 Department of Plant Pathology, University of Agriculture, Faisalabad 38040, Pakistan; 2 Centre of Agricultural Biochemistry and Biotechnology, University of Agriculture, Faisalabad 38040, Pakistan; 3 Institute of Molecular Biology & Biotechnology, Bahauddin Zakariya University, Multan 66000, Pakistan; germplasm@nate.com; 4 Department of Biotechnology, Chonnam National University, Yeosu 59626, Korea; amjad_ucauos@yahoo.com; 5 School of Life Sciences and Centre for Soybean Research of the Partner State Key Laboratory of Agrobiotechnology, The Chinese University of Hong Kong, Shatin, Hong Kong SAR, China; honming@cuhk.edu.hk

**Keywords:** plant parasitic nematode, PPN, nematode effector protein, nematode-associated molecular pattern, NAMP, plant defense signaling pathway, NAMP-triggered immunity, NTI

## Abstract

To successfully invade and infect their host plants, plant parasitic nematodes (PPNs) need to evolve molecular mechanisms to overcome the defense responses from the plants. Nematode-associated molecular patterns (NAMPs), including ascarosides and certain proteins, while instrumental in enabling the infection, can be perceived by the host plants, which then initiate a signaling cascade leading to the induction of basal defense responses. To combat host resistance, some nematodes can inject effectors into the cells of susceptible hosts to reprogram the basal resistance signaling and also modulate the hosts’ gene expression patterns to facilitate the establishment of nematode feeding sites (NFSs). In this review, we summarized all the known signaling pathways involved in plant–nematode interactions. Specifically, we placed particular focus on the effector proteins from PPNs that mimic the signaling of the defense responses in host plants. Furthermore, we gave an updated overview of the regulation by PPNs of different host defense pathways such as salicylic acid (SA)/jasmonic acid (JA), auxin, and cytokinin and reactive oxygen species (ROS) signaling to facilitate their parasitic successes in plants. This review will enhance the understanding of the molecular signaling pathways involved in both compatible and incompatible plant–nematode interactions.

## 1. Introduction

Parasitic nematodes are economically important pests of crop plants and can lead to significant yield losses worldwide [1]. Sedentary endoparasitic nematodes belonging to the family *Heteroderidae* include root knot nematodes from the genus *Meloidogyne* and cyst-forming nematodes from the genera *Heterodera* and *Globodera* [2]. These nematodes have developed very sophisticated mechanisms of parasitizing host plants, which involve the induction of specialized feeding sites in plant roots. These feeding structures are known as giant cells in the case of root knot nematodes and syncytia in cyst-forming nematodes, respectively [3]. Plant parasitic nematodes (PPNs) induce a series of morphological, biochemical, and molecular alterations in the cells of plant roots to facilitate the establishment of these feeding sites [4]. These events are largely affected by nematode secretions through their stylets, setting off a signaling cascade in the host cells.

The first signaling event in plant–nematode interactions involves the movement of second-stage juveniles (J2) towards the plant roots through the process of chemotaxis mediated by the root exudates of the host plants [5]. In the absence of resistance responses from the plant, e.g., production of reactive oxygen species (ROS) and callose deposition, PPNs enter the root cells and locate the vascular tissues for the initiation of feeding sites [6,7]. These nematode feeding sites (NFSs) become the sole nutrient sources for the nematodes as they develop through subsequent sedentary life stages [8,9].

The initiation of NFSs is brought about by nematode secretions that contain cell wall–degrading enzymes (CWDEs), virulence proteins (Avr proteins), and transcription factors [4]. The biochemical analyses of secretions from different PPNs demonstrated a variety of effector proteins that could interact with host genes and proteins to establish successful parasitism [4,10,11]. It has been suggested that the elements in the nematode stylet secretome led to significant changes in the host’s novel gene regulatory cascades, triggering the differentiation of the parasitized root cells into unique feeding cells [12]. Similarly, effectors secreted by the nematodes could reprogramme cellular events in the host plant for the induction of NFSs as well as suppress host defense responses by directly manipulating plant metabolic and developmental pathways [4,11].

PPNs can induce several signaling pathways through nematode-associated molecular patterns (NAMPs) to enable compatible interactions with the plants [13]. Similarly, secretions released by the nematodes can set off different signaling cascades which lead to the activation of plant genes helpful for the establishment of PPNs in plants. At the same time, as a counter measure, the detection of these NAMPs by the host plants also activate the basal immune system to safeguard the plants against nematode invasion [14]. It has also been proposed that as a one-upmanship, nematodes could produce effector proteins to induce changes in the plant’s cell cycle, cytoskeleton, and small RNA production to circumvent the host’s basal defense against parasitism [15,16,17,18]. Furthermore, PPNs are able to modulate different signaling pathways in their hosts, such as gene silencing pathways, jasmonic acid-salicylic acid (JA-SA) pathways, gibberellin (GA) pathways, cytokinin pathways, and post-transcriptional modifications [4,16,19,20,21].

In this review, we have focused on the most recent literature on plant signaling events involved in compatible and incompatible plant nematode interactions, starting from the initial nematode infection to the establishment of the feeding site in the plant root. This review will improve the understanding of various signal transduction pathways involved in plant–nematode molecular interactions by summarizing the most recent discoveries on these topics.

## 2. Cellular Signal Transduction in Plants

For millennia, plants and pathogens are continuously engaged in co-evolution in the struggle for dominance between hosts and pathogens. The invading pathogens face specific multi-layered defense responses generated by the host plant cells. The plant cell wall is the first physical barrier encountered by pathogens including plant parasitic nematodes (PPNs), fungi, bacteria, and viruses. Once the physical barrier is crossed by the pathogen, the host cytoplasm serves as the battleground where a war is fought between host and pathogen molecules. Next to the cell wall, there are membrane-localized pattern recognition receptors (PRRs) that recognize often-conserved pathogen/microbe-associated molecular patterns (PAMPs/MAMPs) such as proteins, lipids, carbohydrates, and cell wall derivatives [22]. As a consequence of the recognition of PAMPs/MAMPs, PRRs initiate a conserved downstream cellular signaling cascade referred to as PAMP-triggered immunity (PTI) inside the cytoplasm of the host cell [22,23]. The responses include the activation of mitogen-activated protein kinases (MAPKs), the production of reactive oxygen species (ROS), and the induction of signaling pathways via salicylic acid (SA) and jasmonic acid (JA) [24,25,26,27]. In plants, all the PRRs are transmembrane proteins containing a ligand-binding extracellular domain. The PRRs are divided into two groups: the receptor-like kinases (RLKs), which contain an intracellular kinase domain for cytoplasmic signaling, and the receptor-like proteins (RLPs), which lack any apparent cytoplasmic signaling domain [22,28]. It is assumed that RLPs always function in association with one or more RLKs for ligand-specific intracellular signal transduction [29]. PTI-triggered immunity usually protects plants against non-adapted microorganisms [30].

The pathogens that are successful in gaining access to the interior of the host cell release effector proteins to interfere with the host immune response (PTI) and to compromise the plant basal defense system. However, this triggers another mode of signal perception in the host by intracellular nucleotide binding/leucine-rich repeats (NLR) receptors [27,30], which induces a robust and strong response, often leading to localized cell death, thus preventing the spread of the disease beyond the initial site of infection. This defense mechanism is referred to as effector-triggered immunity (ETI). The response leading to cell death is generally referred to as hypersensitive response (HR), which also activates the long-lasting resistance in non-infected tissues as well (termed systemic acquired resistance) [22,27,30]. Nevertheless, plant basal immunity comprises two main responses: PTI and ETI [13,31,32]. These plant immune responses largely match the zig-zag model [32] in which molecular pattern release by PPNs could induce defense responses such as NTI (see Section 4), and nematode effector proteins may interact with host R-proteins to prompt ETI followed by localized HR (see Section 8 and Section 10). Plant immunity against PPNs could also be described on the basis of a multicomponent model [33] in which immunity activation component (IAC) is primarily comprised of NAMPs triggered signaling, followed by modification of gene expression of defense related genes by effector proteins (reviewed in [4]), and ultimately vertical immunity brought about by the interaction of R proteins and their corresponding Avr proteins. The immunity modulation component (IMC) involves the development of hyper-metabolic NFSs and modulation of various signaling cascades by different phytohormones [16].

## 3. Nematode-Associated Molecular Patterns (NAMPs) and Signaling for Nematode Resistance

Several studies have shown that plants generate PTI and ETI responses, such as the activation of mitogen-activated protein kinases (MAPKs), apoplastic ROS bursts, and JA and SA signaling, when they encounter nematode infections [34,35,36,37,38]. The first detailed study on NAMPs highlighted the perception of nematode-produced ascarosides by plant cells, triggering the gene expressions associated with MAMP-triggered immunity and the activation of MAPKs as well as JA and SA signaling [37]. Ascarosides are small molecules acting as pheromones in the social behavior of nematodes. In spite of diverse nematode phylogeny and ecology, ascaroside biosynthesis and signaling are highly conserved among nematodes [39]. In *Caenorhabditis elegans* (a free-living nematode) and many other species, ascarosides are involved in social signaling, dauer development, finding mating partners, and coordinating nematode behaviors [39,40,41]. Ascarosides are secreted by nematodes into their environment. Structurally, they are derivatives of the 3, 6-dideoxy-l-sugar, ascarylose, modified with fatty acid-derived side chains, and they are classified according to the number of carbons in their side chains [39,40,41]. Ascr#18 featuring an 11-carbon side chain is the most abundant among three genera of plant parasitic nematodes including root knot and cyst-forming nematodes. The priming of *Arabidopsis* roots with ascr#18 induced general resistance against bacteria, fungi and nematodes, as well as systemic resistance in the leaves of the plant [37,42]. All this evidence indicates that ascarosides secreted by nematodes are possible NAMPs perceivable by plants for the induction of basal defense mechanisms. However, evidence that ascarosides can be recognized by cell surface-localized receptors in plants is still lacking.

Recently another study claimed that the proteinaceous nature of potential NAMPs is a contributing factor in inducing basal immunity in *Arabidopsis* [43]. The authors prepared “NemaWater” by incubating pre-infective-stage juveniles of *Heterodera schachtii* and *Meloidogyne incognita* in water for 24 h. It was assumed that potential NAMPs present on the nematodes’ surface would be dissolved in the water during incubation. Then *Arabidopsis* roots were treated with NemaWater and produced apoplastic ROS bursts, which are hallmarks of plant basal immunity. Meanwhile, the *Arabidopsis* roots treated with NemaWater pre-incubated with proteinase K had significantly reduced ROS bursts. It was pointed out that one or more proteins of nematode origin could have been recognized by the plant cells for the activation of plant immunity. However, the question remains whether such proteins are secreted by pre-infective-stage juveniles on their surfaces. Furthermore, this study showed that the above-mentioned PTI responses were mediated through a membrane-localized leucine-rich repeat receptor-like kinase, *NILR1*, which was specifically induced upon nematode infection [43]. Therefore, it was the first-ever report on *NILR1* as the PRR for perceiving a protein of nematode origin and inducing plant basal immunity against nematode infection. However, further studies are still needed for understanding the downstream signaling of *NILR1*.

Besides ascarosides and nematode-derived proteins, chitin and cuticle are also potential candidates for NAMPs. Chitin is present in the eggshells of various PPNs but absent in plant cells. Cuticle forms the exoskeleton of the nematode and is very important for its movement and growth [13]. However, the roles of chitin and cuticle have not yet been revealed in the induction of plant basal immunity against nematode infection. On the other hand, exudates released by plant roots are important for egg hatching, nematode chemotaxis towards plants, and trigger early events of signaling between plants and nematodes [44]. For instance, root exudates such as eclepins play pivotal roles in egg hatching of cyst nematodes while others could trigger the chemical mediated movement of nematode towards plant roots.

## 4. Phytoalexin Pathway and NAMP-Triggered Immunity (NTI)

**Figure 1:**
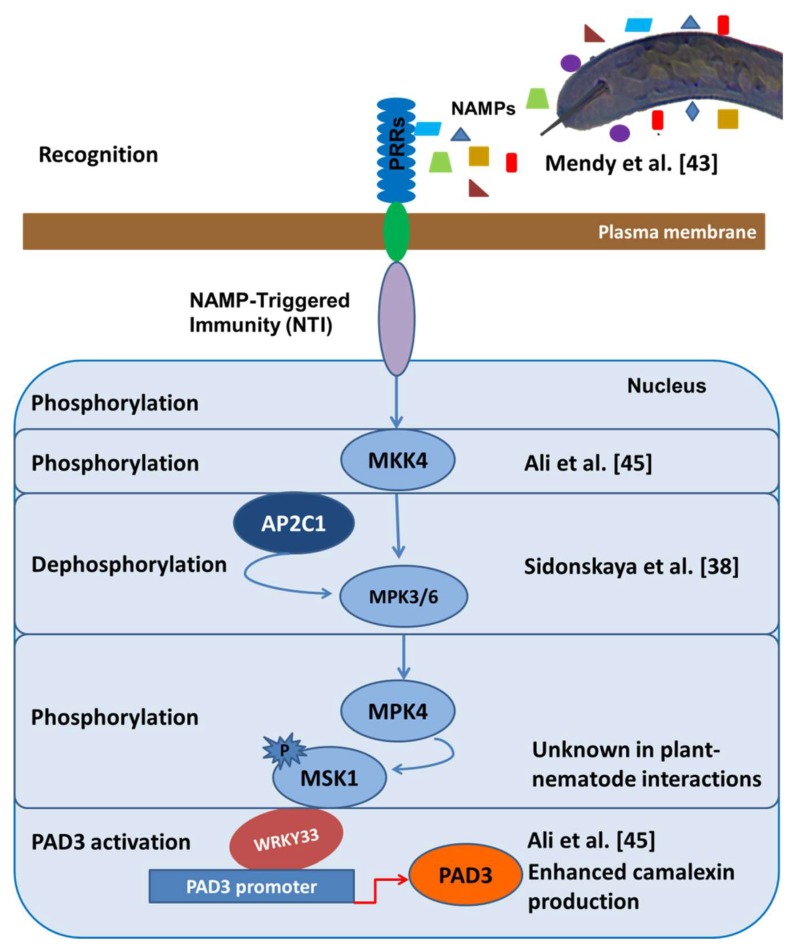
Hypothetical overview of nematode-associated molecular pattern (NAMP)-triggered immunity (NTI) in the *Arabidopsis*-beet cyst nematode *H. schachtii* model system. NAMPs are specific molecular patterns released by plant parasitic nematodes which are recognized by transmembrane receptors such as kinases on the host to induce a signaling cascade [13]. The overexpression of *MKK4* in *Arabidopsis* led to increased resistance against beet cyst nematode as compared to wild type, suggesting an important role of *MKK4* in signal transduction in plant immunity [45]. MAPK phosphatase *AP2C1* may be involved in the putative dephosphorylation of *MKK* (MAP kinase kinase) into the *MPK3* and *MPK6* kinases to further transduce the signal for nematode resistance [38]. We have experimentally demonstrated that the overexpression of *WRKY33* and the knocking-out of *PAD3* resulted in enhanced nematode resistance, which highly support our hypothesis that the phosphorylation cascade involving *WRKY33* leads to camalexin biosynthesis which in turn leads to NTI [45]. Although some steps, such as the phosphorylation of *MKK4* and *MSK1* and the activation of *WRKY33*, in this cascade have not yet been demonstrated in plant–nematode interactions, the proposed NTI model indicates the importance of *WRKY33*-dependent camalexin production in *Arabidopsis*.

PPNs have the ability to suppress important genes involved in the early events of defense responses in plants. Phytoalexins are important defense-related plant metabolites in the initial stages of plant–nematode interactions [45,46]. The biosynthesis of these metabolites is a key component of the innate immune system and involves a series of signaling events. The transcription factor, *WRKY33*, is considered to be the key player in the activation of camalexin-based PTI in plants [47,48]. This type of PTI comprises signal perception by transmembrane receptors followed by the commencement of signaling cascades through various MAPKs such as MPKs, MKKs, MKKKs, etc., involving several phosphorylation and dephosphorylation events [49]. These signaling cascades lead to the upregulation of the *WRKY33* protein that in turn activates the *PAD3* gene to induce camalexin production [47]. As supporting evidence, *WRKY33* was the most downregulated WRKY transcription factors in the syncytia induced by *H. schachtii* compared to uninfected *Arabidopsis* roots [45,50]. *MKK4* was shown to be the primary signaling kinase that activated *WRKY33* and camalexin-mediated PTI. The overexpression of both *WRKY33* and *MKK4* resulted in enhanced resistance against *H. schachtii* in *Arabidopsis* while a T-DNA-insertion mutant of *PAD3* led to increased susceptibility [45]. However, the phosphorylation and dephosphorylation events in this signaling cascade have not yet been worked out completely (Figure 1).

## 5. Nematode Effector Proteins

All PPNs contain a distinctive structure known as a stylet that is employed for puncturing plant cell walls, easing the movement of PPNs into the plant tissues, and for the subsequent injection of esophageal secretions into the host cells to induce NFSs [51]. However, a less significant amount of secretion is also released through the amphids and the cuticle of the nematode [4]. The stylet secretions contain a large number of effector and parasitism proteins which are developmentally controlled in different parasitic life stages of PPNs [51,52,53]. The secretions are collectively called the “secretome,” and the secreted proteins indirectly or directly involved in the development of compatible interactions with plant hosts are collectively known as the “parasitome” [2]. These are the secretions released by the nematode that facilitate the differentiation of host root cells into sophisticated and complex NFSs [12,51,54].

The secretomes of sedentary endoparasitic nematodes are particularly fascinating due to their ability to cause major alterations in host gene expressions. This altered expression pattern leads to metabolic, biochemical and morphological changes in infected root cells to turn these cells into unique NFSs [12]. The nematode secretome comprises highly concentrated proteins with huge diversity originating from divergent gland cell types [54,55]. The first complete parasitome profiling was carried out in the soybean cyst nematode (SCN), *Heterodera glycines* [56]. Similarly, the secretome of a root knot nematode, *Meloidogyne incognita*, consisted of 486 proteins [57]. Interestingly, a significant number of these secreted proteins from nematode secretions are homologues of plant proteins that could be stimulated by the nematode secreted proteins to induce plant growth and cell cycle reprogramming of gene expressions in the host cell [57].

The nematode parasitome primarily contains three categories of proteins [4]. The first category includes cell wall-degrading enzymes (CWDEs) and cell wall modification enzymes for localized cell wall degradation to facilitate the movement of PPNs through the plant root tissues. The second category of secreted proteins consists of nematode effectors that are involved in the modification of metabolic events and virulence factors that interact with resistance (R) proteins from the plant host, leading to enhanced resistance and/or susceptibility. The last category is the regulatory proteins that bind to the promoters of plant genes to modulate their expressions in favor of PPNs and the establishment of NFSs. These regulatory proteins are therefore transcription factors which act either as activators of plant genes helpful for the induction of NFSs, e.g., ATPases [58], or, more frequently the case, as suppressors of host genes involved in resistance responses, e.g., *AtWRKYs* and *AtRAP2.6* [45,46].

The secreted proteins are either deposited outside the plasma membrane of the root cells or injected into the cytoplasm of the infected cells. In both situations, particular elicitors present in the nematode secretions could bind to plant cell receptors to induce a signal transduction cascade for the modification of host gene expressions [59,60,61].

## 6. CLE Signaling and Parasitic Success of Nematodes

**Figure 2:**
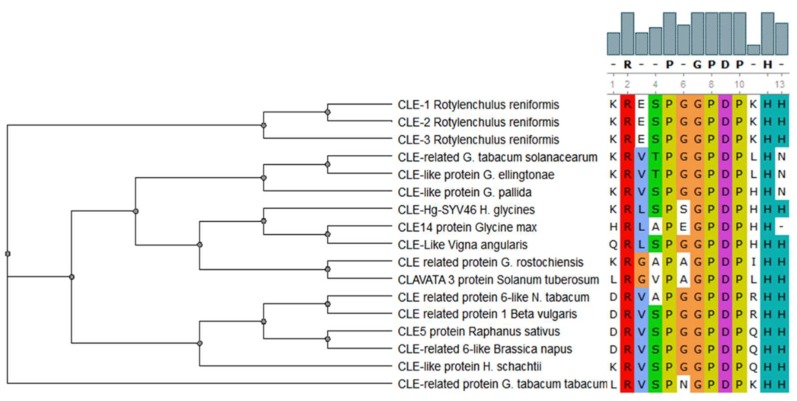
Conservation of the CLE motif (xRxxPxGPDPxHx) in CLAVATA/ESR (CLE) peptides in different plant parasitic nematodes (PPNs) and plant species [4]. This multiple alignment demonstrates the highly conserved amino acid residues of CLE motif between PPNs and their corresponding host plants.

CLAVATA/ESR (CLE) peptides are involved in both the promotion and the inhibition of cell differentiation in meristematic tissues in plants, and members of this family of signaling peptides are also present in PPNs [61]. CLE peptides contain two highly conserved motifs: KRLVPSGPNPLHH and LxLxxxLILxLLLxS [62]. The *CLE* motifs from various PPNs are very similar to the CLE motifs found in their corresponding host plants [4] (Figure 2). This suggests that *CLE* peptides from PPNs are possible stimulators of cell differentiation and/or proliferation in host plants. This is why the establishment of syncytia and the differentiation of vascular tissues, e.g., xylem, so closely resemble each other [63]. There is a detailed review regarding the involvement of CLE signaling in plant development and plant–nematode interactions [64].

It has been demonstrated that receptor kinases like *CORYNE (CRN)* and *CLAVATA2 (CLV2)* are essential for CLE signaling for compatible plant–nematode interactions and the establishment of syncytia induced by *H. schachtii* in *Arabidopsis* [65]. The receptor kinases such as *CLV1* and RECEPTOR-LIKE PROTEIN KINASE 2/TOADSTOOL2 (*RPK2*) are primarily involved in the maintenance of shoot apical meristem in *Arabidopsis*. These two receptors are also able to transmit the *CLV3*-independent signaling of *CLV2/CRN* upon CLE perception for the parasitic success of PPNs in plants [66]. Similarly, the CLE peptide from *G. rostochiensis*, *GrCLE1*, was glycosylated into a 12-amino acid arabinosylated glycopeptide resistant to hydrolytic degradation and with high-affinity binding to the potato *CLV2*-like receptor (*StCLV2*) [67]. This shows that the process of glycosylation is important in the CLE signaling process for compatible plant–nematode interactions. Moreover, the processing of *GrCLE1* by host plant proteases enables this peptide to directly bind to the plant CLE receptors *CLV2*, *BAM1*, and *BAM2*, to modulate their functions [68]. CLE peptides also interact with plant genes to establish NFSs in roots [69]. A new class of CLE peptides known as B-type CLE peptides from *Arabidopsis* was found to involve in TDIF (tracheary element differentiation inhibitory factor)-TDR (*TDIF* receptor)-*WOX4* pathway to induce the development of NFSs [70]. Primarily, this pathway is involved in proliferation of procambial meristematic cells. However, it was demonstrated that the TDIF pathway could be activated in syncytia induced by *H. schachtii* in *Arabidopsis* roots [70]. Loss-of-function mutants of various genes involved in this pathway (*cle41, tdr-1, wox4-1*, and double mutant *tdr-1 wox4-1*) exhibited lower number of nematodes along with compromised development of syncytia. These reports showed that CLE peptides are important in the developmental events in the meristem and are also involved in CLE signaling for the promotion of nematode parasitism in plants.

## 7. Nematode Effectors Are Targeted to Plant Cell Nuclei to Manipulate Host Functions

PPNs are able to alter various signaling pathways in their hosts with the help of effectors. For instance, 10A06 from *H. schachtii* modulates the biosynthesis of spermidine, which leads to enhanced antioxidant protection coupled with the interruption of salicylic acid (SA) signaling [71]. Some nematode effectors are localized in the plant cell nucleus and act as transcriptional activators or suppressors of plant genes. Recently, an *M. incognita* effector protein, Mi-EFF1, was found in the nuclei of the multinucleate NFSs and was postulated to be involved in the manipulation of gene expressions of host cells [72]. Nonetheless, the precise function of this effector is still unknown. Similar subcellular localization was observed for a novel *M. javanica* effector, *Mj-NULG1a*, which was targeted to the nuclei of giant cells to induce compatible interactions with the host [73]. Similarly, a *M. incognita* calreticulin, *Mi-CRT*, was found in the apoplasm of plant cells and was a key player in the suppression of basal defense of infected *Arabidopsis* plants [74,75,76]. This suppression in basal defense was demonstrated in the *Mi-CRT* overexpression lines by the downregulation of genes involved in inducing JA-SA signaling and callose production following the application of a PAMP, elf18 [76]. The homologous protein from *B. xylophilus*, *Bx-CRT1*, was found to be important for the differentiation of NFSs and cell-to-cell trafficking [77]. Similarly, the 10A07 effector from *H. schachtii* was found to recruit Interacting Plant Kinase (*IPK*) and the *IAA16* transcription factor for its phosphorylation at serine-144 and 231 to mediate its trafficking from the cytoplasm to the nucleus [78].

The beet cyst nematode was shown to secret an ANNEXIN-like protein, *Hs4F01*, with high sequence similarity to plant annexins, and able to mimic plant annexins during the parasitic interaction, through binding phospholipids and calcium, which are important modulators of plant defense [79]. However, a recent report demonstrated that Ha-annexin from cereal cyst nematode *H. avenae* was targeted to the plant nucleus to suppress basal defense responses of the host [80]. Stable silencing of this gene led to abnormal nematode development on wheat roots, whereas transient expression of Ha-annexin resulted in the suppression of programmed cell death (PCD) induced by different PAMPs and the *BAX* protein [80]. Similarly, silencing and overexpression of *MiMsp40* in *Arabidopsis* led to the suppression and establishment of parasitism by *M. incognita*, respectively, through the respective enhancement and suppression of PAMP-triggered immunity and callose deposition [81]. In this case, both PTI and ETI were reduced, through the interactions between *BAX* and cognate elicitors R3a/Avr3a to inhibit HR and facilitate nematode establishment in the plant root [81].

Among different types of effector proteins released by the nematodes, transcription factors are very important players involved in modulation of host gene expression [4]. A total of 46 differential transcription factor binding sites (TFBSs) in soybean lines with contrasting resistance against soybean cyst nematode, *H. glycines* were identified [82]. *HAHB4*, *DOF*, *MYB*, and *ARF* boxes are some of the important examples of these differentially represented TFBSs [82]. The authors proposed that these over–represented TFBSs in soybean could play important roles in *cis*-regulatory dynamics.

## 8. Nematode Effectors Mimic Defense Responses in Host Plants

At the same time that effector proteins originating from PPNs are mimicking various plant proteins and suppressing plant defense responses, their perception by the host plant would induce host defense against their invasion. The very first step of these responses is the recognition of nematode effectors (Avr proteins) by resistance (R) proteins from the host plant, leading to the activation of ETI [4]. PPNs contain numerous Avr proteins that result in gene-for-gene interactions and play a central role in incompatible plant–nematode interactions. Important examples of these Avr proteins are *SPRYSEC-19* [83,84], *map-1.2* [85], chorismate mutase from *H. glycines* (*Hg-cm-1*) [85,86,87], *Cg-1* from *M. incognita* [88], and *Gr-VAP1* [89] and *Gp-Rbp-1* [90,91] from pale and white potato cyst nematodes, respectively.

*Cg-1* and *map-1.2* proteins interact with the *Mi-1* R protein, a member of CC-NBS-LRR (CNL) class of R-proteins, to induce Mi-1-mediated ETI in tomato plants [85,88,92]. Experimental evidence shows that *SPRYSEC-19* from *G. rostochiensis* is recognized by the leucine-rich repeat (LRR) motif of the tomato CNL family R protein, *SW5-F* [83]. However, surprisingly, *SPRYSEC-19* did not induce PCD in tobacco leaves when transiently co-expressed with the SW5-F protein [84]. This suggests that *SPRYSEC-19* somehow suppresses CNL-mediated resistance responses in plants. Conversely, a SPRYSEC gene from *G. pallida* (*Gp-Rbp-1*) led to the activation of HR after it was co-expressed with the potato resistance genes, *Gpa2* and *RanGAP2* (*Ran GTPase Activating Protein 2*) [90,91]. The variability in the amino acid sequence of potato RanGAP2 is a main contributing factor for the recognition of Avr proteins by *Gpa2* to enhance nematode resistance in potato [93].

The venom allergen-like proteins (Vap) are also important PPN components recognized by host plants to mount a defense response against the nematode infection [89,94,95,96]. A strong interaction was demonstrated between the *G. rostochiensis* Vap protein (*Gr-Vap1*) and the tomato cysteine protease, *Rcr3pim*, and immune receptor protein, *Cf-2*, to induce PCD and enhanced resistance against the infection by *G. rostochiensis* [89,97]. It seems that both of these genes in tomato are involved in signal perception and interaction with unknown R proteins to confer resistance responses. As *Gr-VAP1* has also been shown to activate basal innate immunity in the potato hairy roots following the infection with *G. rostochiensis*, it may be regarded as a PAMP to elicit PTI as well. Another effector protein from *G. rostochiensis*, *GrEXPB2*, can both suppress and elicit defense responses in the plant. On one hand, this effector is involved in the suppression of PCD induced by a *Phytophthora infestans* extracellular elicitor, *PiNPP*, and NB-LRR mediated immune responses by the inhibition of an immune receptor of *Rx* protein in tobacco leaves [98]. On the other hand, *GrEXPB2* was also found to elicit defense responses in a number of potato and tomato lines [98].

It has been reported that the nematode effector protein, *Mj-FAR-1*, from *M. javanica* could be involved in the manipulation of lipid-based molecular signaling and inhibition of JA-mediated signaling in plant–nematode interactions [99]. Likewise, similar studies have also demonstrated the participation of different effector proteins in both the suppression and activation of basal immune responses of the host plants [100]. Overall, the majority of reported effectors function by manipulating the hosts’ nuclear functions to develop NFSs [4].

In addition to well characterized *R* genes, a few quantitative trait loci (QTLs) regulating nematode resistance were also reported. Among them, *Cre* and *Rhg* are well known examples in wheat and soybean conferring resistance against cereal cyst nematodes and soybean cyst nematodes (SCN) respectively. *Rhg1* and *Rhg4* are naturally occurring QTLs identified in soybean that control resistance responses against SCN [35,101]. Experimental evidence showed that *Rhg4* encodes a serine hydroxymethyltransferase and overexpression of this enzyme in soybean roots led to 45% reduction in the number of mature cyst nematodes [102]. Genome-wide association study (GWAS) could also be an effective way to identity resistance QTLs. For instance, 13 single nucleotide polymorphisms (SNPs) associated with soybean cyst nematode resistance in soybean were identified, in which 3 SNPs were linked to the previously reported QTLs *Rhg1* and *Rhg4* [103].

## 9. Roles of SA/JA, Auxin, and Cytokinin Signaling in Plant–Nematode Interactions

Plant hormones such as auxins and cytokinins are important in plant–nematode interactions because they are involved in the establishment of NFSs in plant roots [21,104,105]. SA and JA are key players involved in the activation of different signaling pathways in defense responses in plants [19,20,34]. Callose production is one of the earliest defense responses of plants against the invading PPNs [9]. Overexpression of an *Arabidopsis* ethylene-responsive transcription factor, RAP2.6, led to enhanced production of JA and callose in NFSs as compared to control plant roots. However, resistance in tomato against *M. incognita* is primarily associated with SA signaling instead of the typical JA signaling [19,20]. In the same way, *HeroA*-based resistance responses in potato involves elevated expressions of SA-induced *PR* genes following the infection by potato cyst nematode, while in susceptible potato lines, the SA signaling pathway was inhibited [106]. Comparable results were found in a resistant wheat line expressing the *Cre2* gene against cereal cyst nematode, *H. avenae* [107].

Nematode effector proteins can modulate auxin-based signaling and defense responses. For instance, the *H. schachtii* effector, 19C07, can bind the *Arabidopsis* auxin transporter, *LAX3*, to control auxin influx and the formation of syncytia [108]. Likewise, a chorismate mutase-coding gene from *M. javanica*, *MjCM-1*, regulates auxin signaling through altering the levels of indole-3-acetic acid (IAA) in host cells, which leads to alterations in chorismate metabolism as well as chorismate-derived metabolites to promote cell enlargement and the induction of NFSs [109]. Moreover, the upregulation of auxin response transcription factors (ARFs) in NFSs suggests the putative role of auxin signaling in compatible plant–nematode interactions [110]. By studying the transcriptome of *H. glycines*-resistant soybean lines, it has been demonstrated that auxin-induced biosynthesis of ROS is associated with increased Ca^2+^ conductance across the cell membrane in the resistant lines. Furthermore, DELLA-like proteins are probably involved in auxin-regulated ROS signaling to produce resistance responses in soybean [111].

Cytokinins are required for the successful development of syncytia in *Arabidopsis*. This was discovered by infecting cytokinin-deficient *Arabidopsis* mutants with *H. schachtii*. The results revealed decreased susceptibility of cytokinin-deficient mutants against the PPN [112]. It was also reported that isopentenyl transferase, the key player involved in the biosynthesis of cytokinin, could modulate nematode virulence, the division of host cells, and the establishment of NFSs in *Arabidopsis* roots [112].

Recently, the expressions of various genes involved in the defense mechanisms in resistant versus susceptible rice cultivars were compared after infection with *M. graminicola* [113]. The expressions of several genes involved in lignin biosynthesis and callose deposition were enhanced in the resistant cultivar compared to the susceptible one. Moreover, MAPKs such as *OsMAPK5*, *OsMAPK6* and *OsMAPK20* were activated in the early stages of *M. graminicola* infection in a nematode-resistant rice cultivar [113]. Furthermore, SA-JA as well as ethylene-related genes in the resistant line were also activated, suggesting the activation of various defense mechanisms involving these pathways in rice. Similar results were shown in wild soybean–*H. glycines* interactions in which several MAPKs and SA-JA signaling genes were upregulated during resistance responses [114]. More recently, two transcription factors, *DAF-16* and *SKN-1* from various free-living and parasitic nematodes, were identified as key players involved in important signaling pathways including SA, JA, MTI, and ROS biosynthesis [115].

## 10. Parasitic Nematodes Modulate ROS Signaling and PCD in Plants

Programmed cell death (PCD) and hypersensitive response (HR) are the main results of ROS signaling in plants. Plants are able to program the suicidal death of their cells upon pathogen invasion through the biosynthesis of ROS. This leads to HR that restricts the pathogen’s spread through living tissue and kills the pathogen at the site of infection. However, the biosynthesis of ROS is largely reliant on the accumulation of SA at the site of infection [116,117]. It has been reported that the production of ROS is one of the earliest events in molecular plant–nematode interactions [9]. R proteins frequently lead to the upregulation of genes involved in ROS production which results in HR. Soybean lines harboring the *Rhg1* gene displayed elevated expressions of ROS-related genes and enhanced resistance to soybean cyst nematode, *H. glycines* [35]. Moreover, DELLA-like proteins were found to be involved in auxin-induced signaling for the biosynthesis of ROS in soybean lines resistant to *H. glycines* [111].

As mentioned earlier, higher concentrations of SA stimulate the biosynthesis of ROS and result in PCD in plants [118,119]. However, the SA-dependent ROS biosynthesis pathway and the ROS biosynthesis pathway mediated by Respiratory Burst Oxidase Homologs (RBOHs) are mutually antagonistic in *Arabidopsis* [120]. It has been reported that PPNs can manipulate the RBOH-dependent ROS pathway for parasitic success in plants. This study demonstrated that *H. schachtii* was able to stimulate the expression of *RBohD* and *RBohF* in *Arabidopsis* to induce the synthesis of ROS that surprisingly resulted in the inhibition of PCD and therefore supported the development of syncytia [36].

Plants have a variety of enzymes (e.g., peroxidases) to scavenge excessive ROS in the plant tissue to prevent self-damage. In the same way, secretomes of PPNs contain superoxide dismutases (SODs) to detoxify ROS produced by the plant cells and dampen resistance responses [57,121]. Similarly, PPNs can scavenge the excess ROS with enzymes and antioxidants, such as glutathione, peroxidases, thioredoxins, cytochrome C-peroxidases, ascorbate peroxidases, and catalases, which protect the nematode cells from ROS-mediated injuries [57,122]. Additionally, the overexpression of a peroxiredoxin-family gene from potato cyst nematode, *G. rostochiensis*, protected it against defense responses from the potato plant through an array of redox reactions [123].

## 11. Nematodes Modulate RNA Silencing Pathways to Promote Infection Process

RNA silencing is an important strategy of plant responses against abiotic and biotic stresses, in which microRNAs (miRNAs) are important modulators [124]. Using deep sequencing to compare miRNA profiles, 60 differentially expressed miRNA from 25 diverse miRNA families associated with responses against SCN were identified [125]. Moreover, an *Arabidopsis* microRNA, mi396, was reported to interact with transcription factors like Growth-Regulating Factor, GRF1/GRF3 to modulate the reprogramming of root development under *H. schachtii* infection [126]. The same technique was used to analyze miRNAs in soybean after infection with SCN, which led to the identification of 20 miRNAs with diverse expression pattern in resistant and susceptible soybean cultivars [127]. It has been shown recently that the majority of these miRNAs were predominantly upregulated in susceptible cultivars as compared to the resistant counterparts [128]. The expression of these upregulated miRNAs (i.e., *miR156*, *miR159*, *miR164*, and *miR396*) was found to be negatively correlated with the expression of their target transcription factors such as SBP, GAMYB-like, NAC, and *GRF1*, respectively. These studies suggest a pivotal role of miRNAs in the regulation of regulatory transcription factors responsible for plant resistance responses.

## 12. Conclusions and Future Perspectives

PPNs are able to induce and modulate different signaling pathways in plants with the help of their effector proteins. These effectors can not only induce the auxin and cytokinin signaling for the development of NFSs but can also suppress the SA and JA signaling to avoid host defense responses. PPNs release NAMPs that lead to the signaling for camalexin-based NTI in plants. Similarly, PPNs could regulate different defense pathways, e.g., ROS production, to their own advantage to establish compatible interactions with the plant hosts. *In-planta* RNA silencing of genes from PPNs as well as those of host plants involved in various signaling pathways could be helpful for understanding molecular signaling in plant–nematode interactions [129]. The use of recently developed transcriptomic technologies such as high-throughput RNA-seq will be helpful for the identification of interacting genes in various signaling pathways related to plant defense mechanisms [114,130]. The secretomes of PPNs and transcriptomes of numerous host plants reveal a significant number of differentially regulated plant genes [4], which could be potential candidates for understanding the site-specific silencing in syncytia to enhance nematode resistance in plants [131].

Emerging molecular technologies such as deep sequencing could be of potential use for high throughput studies to provide a holistic view of signaling events involved in the regulation of plant nematode interactions [114]. Gland cell RNA sequencing could be exploited to understand the functioning of various effectors associated with signaling process [132]. Similarly, genome editing approaches like CRISPR-Cas9 could be employed for exploring the function of various plant and nematode genes involved in compatible and incompatible plant–nematode interactions. These genome editing technologies (GETs) could be helpful in understanding the interaction of nematode effector proteins with R proteins from plants [133].
